# Quantification of the purinergic P2X_7_ receptor with [^11^C]SMW139 improves through correction for brain-penetrating radiometabolites

**DOI:** 10.1177/0271678X221126830

**Published:** 2022-09-26

**Authors:** Joachim Brumberg, Richard Aarnio, Anton Forsberg, Päivi Marjamäki, Vera Kerstens, Mohammad M Moein, Sangram Nag, Saara Wahlroos, Michael Kassiou, Albert D Windhorst, Christer Halldin, Merja Haaparanta-Solin, Patrik Fazio, Vesa Oikonen, Juha O Rinne, Andrea Varrone

**Affiliations:** 1 Centre for Psychiatry Research, Department of Clinical Neuroscience, Karolinska Institutet & Stockholm Health Care Services, Stockholm, Sweden; 2 Department of Nuclear Medicine, Medical Center – University of Freiburg, Freiburg, Germany; 3 Turku PET Centre, University of Turku and Turku University Hospital, Turku, Finland; 4 School of Chemistry, The University of Sydney, Sydney, Australia; 5 Department of Radiology and Nuclear Medicine, Amsterdam UMC location Vrije Universiteit Amsterdam, Amsterdam, The Netherlands; 6 Amsterdam Neuroscience, Brain Imaging, Amsterdam, The Netherlands; 7 Department of Neurology, Karolinska University Hospital, Stockholm, Sweden

**Keywords:** [^11^C]SMW139 PET, dual input modeling, neuroinflammation, purinergic receptor, radiometabolite

## Abstract

The membrane-based purinergic 7 receptor (P2X_7_R) is expressed on activated microglia and the target of the radioligand [^11^C]SMW139 for *in vivo* assessment of neuroinflammation. This study investigated the contribution of radiolabelled metabolites which potentially affect its quantification. *Ex vivo* high-performance liquid chromatography with a radio detector (radioHPLC) was used to evaluate the parent and radiometabolite fractions of [^11^C]SMW139 in the brain and plasma of eleven mice. Twelve healthy humans underwent 90-min [^11^C]SMW139 brain PET with arterial blood sampling and radiometabolite analysis. The volume of distribution was estimated by using one- and two- tissue compartment (TCM) modeling with single (*V*_T_) and dual (*V*_Tp_) input functions. RadioHPLC showed three major groups of radiometabolite peaks with increasing concentrations in the plasma of all mice and humans. Two radiometabolite peaks were also visible in mice brain homogenates and therefore considered for dual input modeling in humans. 2TCM with single input function provided *V*_T_ estimates with a wide range (0.10–10.74) and high coefficient of variation (COV: 159.9%), whereas dual input function model showed a narrow range of *V*_Tp_ estimates (0.04–0.24; COV: 33.3%). In conclusion, compartment modeling with correction for brain-penetrant radiometabolites improves the *in vivo* quantification of [^11^C]SMW139 binding to P2X_7_R in the human brain.

## Introduction

Inflammatory response to cerebral accumulation of misfolded proteins contributes to disease development of neurodegenerative diseases.^1,2^ Microglia are the main immune cells of the central nervous system and detect changes in their cellular environment, provide physiological function, and promote tissue repair mechanisms.^3^ Whereas initial microglial activation is assumed to have a beneficial effect, chronic and excessive stimuli may result in neuronal damage and thus, trigger the progression of neurodegeneration.^4,5^ The membrane-based purinergic, ligand-gated ion channel 7 receptor (P2X_7_R) is an adenosine triphosphate (ATP)-gated, non-selective cation channel and is upregulated in activated microglia.^6–8^ Whereas it physiologically has trophic actions on neurons, microglia, and astrocytes, it is activated by high ATP-levels after tissue injury, leading to the release of pro-inflammatory substances.^9^ This triggers further microglial activation, promotes neuronal damage, and subsequent apoptosis, that induces a continued ATP-release and thus, sustains a vicious circle of neuronal inflammation and cell loss.^6^ Due to its involvement in this cycle, the P2X_7_R is a promising molecular target to reveal the pathophysiologic mechanisms of neuroinflammation and for new therapeutic approaches.

Four radiolabeled adamantanyl benzamide analogues with high affinity to the human P2X_7_R have been evaluated in rodent animal models, of which [^11^C]SMW139 showed highest binding to the receptor.^10^ The radioligand is not a substrate of the P-glycoprotein at the blood-brain-barrier (BBB) and uptake can be blocked with P2X_7_R antagonists.^11^ [^11^C]SMW139 was evaluated in humans including the characterization of [^11^C]SMW139 pharmacokinetics.^12^ However, the suggested kinetic model did not account for the possible presence of brain-penetrating radiometabolites of [^11^C]SMW139 as indicated by preclinical evidence.^10^ Therefore, this study aims to extend previous findings on brain-penetrating radiometabolites by evaluating [^11^C]SMW139 metabolism preclinically and to assess whether kinetic modeling with dual (i.e. parent and radiometabolite) input functions improves the *in vivo* quantification of P2X_7_R expression in humans.

## Materials and methods

### Small animal study

Eleven C57BL/6J mice were examined with [^11^C]SMW139 (all female; weight: 34 ± 4 g; age: 8.7 ± 1.9 months; injected activity: 17.9 ± 6.2 MBq; molar activity: 42.2 ± 24.1 MBq/nmol; injected mass: 0.21 ± 0.12 µg; for radiochemistry see Supplemental methods ^13,14^). *Ex vivo* sampling for parent fraction analysis was performed at 10 min (*n* = 4), 30 min (n = 3) and 45 min (n = 4) post radioligand injection (p.i.) by cardiac puncture and by collecting perfused brain samples. Sample size of n ≥ 3 per group was considered sufficient for exploratory radiometabolite analysis. All experimental animal procedures were done in accordance with the EU Directive 2010/63/EU on the protection of animals used for scientific purposes, and with the ARRIVE guidelines at Turku PET Centre, University of Turku, Finland [TU] and approved by the Regional State Administrative Agency for Southern Finland (ESAVI/16273/2019).

#### Blood and brain radiometabolite analysis

Blood samples were collected into heparinized tubes (Microtainer, BD, Franklin Lakes, NJ, USA) and centrifuged (12100 g, 90 s). The plasma pipetted into an Eppendorf tube and mixed with 1:2 (v/v) plasma:acetonitrile to precipitate the proteins. The mixture was vortexed for 10 s, centrifuged, and the supernatant (200–700 µL) was collected for subsequent radio detector high-performance liquid chromatography (radioHPLC) analysis. Obtained brains were homogenized in a glass homogenizer with a 9:1 (v/v) acetonitrile:water solution. The homogenized solution was centrifuged (12100 g, 90 s) and the supernatant (200–700 µL volume) was analysed with radioHPLC (see Supplemental methods).

### Human study

Human participants were prospectively enrolled and examined at two study sites (TU and Centre for Psychiatry Research, Karolinska Institutet, Stockholm, Sweden, [KI]). The study was approved by the Ethics Committee of the South Western Finland Hospital District, the Finnish Medicinal Agency, the Ethics Committee of the Stockholm Region, and the Radiation Safety Committee of the Karolinska University Hospital. The study was registered as Clinical Trial in the EudraCT database (2017-001585-19). All human participants provided written, informed consent before participating in the study, which was conducted in accordance with the Declaration of Helsinki and its later amendments. Twelve healthy subjects (TU: seven, KI: five) were recruited through public advertisement. Inclusion criteria were: age between 45 and 80 years, good health according to medical and psychiatric history, physical examination, cognitive assessment (Mini-Mental State Examination ≥ 28), laboratory tests, electrocardiogram, and unremarkable magnetic resonance (MR) imaging of the brain.

#### Imaging procedures

All subjects underwent brain MR scans, including T1-weighted three-dimensional sequences on a 3 Tesla system prior to PET examination as part of the initial evaluation and to delineate anatomic brain volumes of interests (VOI). Six min transmission scans with a ^137^Cs source and the dynamic PET measurements were performed on a High-Resolution Research Tomograph system (Siemens Medical Solutions) at both study sites. [^11^C]SMW139 (424.0 ± 54.8 MBq; 152.0 ± 131.5 MBq/nmol; 1.91 ± 1.31 µg) was injected as bolus into the cubital vein and the catheter was flushed with 10 mL 0.9% NaCl solution. Following injection, emission data were collected in list mode for 90 min. PET data were reconstructed in 21 frames of increasing duration (3 × 5 s, 3 × 10 s, 4 × 60 s, 2 × 150 s, 2 × 300 s, 7 × 600 s) using 3D ordinary Poisson ordered subset expectation maximization (8 iterations, 16 subsets, voxel size of 1.22 × 1.22 × 1.22 mm). Frame-to-frame co-registration of reconstructed images was applied as previously published.^15^

#### Image analysis

All data were processed at KI using an in-house pipeline written in MATLAB (MATLAB r2014b, The MathWorks, Inc.). Individual T1-weighted sequences were segmented with FreeSurfer (FreeSurfer v6.0.0, http://surfer.nmr.mgh.harvard.edu/).^16^ The generated segmentation masks were used to define bilateral VOIs of the frontal cortex, parietal cortex, temporal cortex, caudate nucleus, putamen, thalamus, brainstem, cerebellar cortex, and the whole-brain grey matter. Thereafter, MR and dynamic PET data were co-registered and time-activity curves (TAC) of VOIs were obtained.

#### Arterial blood sampling, radiometabolite analysis, and data processing

A catheter was placed into the radial artery of each participant and arterial blood was collected continuously during the first 5-10 min using an automated blood sampling system (ABSS, Allogg AB). A series of arterial blood samples were drawn manually at ∼2, 5, 10, 20, 40, 60, 75, and 90 min p.i. (Supplemental Table 1). The radioactivity in blood and plasma samples (0.7–1.7 mL) were measured in a well counter cross-calibrated with the PET system. The fraction of unchanged [^11^C]SMW139 (i.e. parent fraction) in arterial blood was measured using radioHPLC analysis. At TU, the same chromatographic method as described for the small animal studies was used. At KI, the plasma was precipitated after centrifugation utilizing a simple protein precipitation method ^17^ and the supernatant was analysed by a reversed-phase radioHPLC as previously described.^18^ The radio-chromatograms for radioactive compounds were integrated and their areas were calculated as a percentage of the total of the decay corrected areas of all detected radioactive fractions. Blood data were processed as previously described with slight modifications to generate plasma TACs,^19^ parent input of [^11^C]SMW139, and one input curve representing the activity of presumably brain-penetrant [^11^C]SMW139 radiometabolites in the plasma (see Supplemental methods).

#### Kinetic modeling

Three kinetic models were assessed to characterize [^11^C]SMW139 kinetics using PMOD (version 3.7, PMOD Technologies LLC). All models were applied by fitting the blood volume (*V*_B_) as an additional parameter.^12^ First, standard two-tissue compartment model with a single (i.e. parent) input curve was assessed with a dual run procedure (2TSI[*k*_4_]), for which the efflux rate from the specific to the non-specific compartment, *k*_4_, was first estimated in the whole-brain grey matter VOI and then used to fix *k*_4_ for subsequent volume of distribution (*V*_T_) estimation in (sub-)cortical grey matter VOIs.^12^ This resulted in four parameters estimated in the second run (*V*_B_, *K*_1_, *k*_2_, *k*_3_). Second, one- and two-tissue compartment models with dual (i.e. parent and radiometabolite) input curves were evaluated (1TDI and 2TDI).^20^ This led to five parameters for 1TDI (*V*_B_, *K*_1p_, *k*_2p_, *K*_1m_, *k*_2m_) and seven parameters to fit for 2TDI (*V*_B_, *K*_1p_, *k*_2p_, *k*_3_, *k*_4_, *K*_1m_, *k*_2m_). In analogy to 2TSI[*k*_4_], the dual input two-tissue compartment model was also assessed with a two-step procedure (2TDI[*k*_4_]; six parameters: *V*_B_, *K*_1p_, *k*_2p_, *k*_3_, *K*_1m_, *k*_2m_). According to the models, the total distribution volumes ^21^ can be achieved by solving equation (1) for 2TSI, equation (2) for 1TDI, and equation (3) for 2TDI where *V*_Tp_ is the distribution volume of the unchanged radioligand. The models assumed that the brain-penetrant radiometabolites did not specifically bind to the P2X_7_R, so that the rate constants *k*_3_ and *k*_4_ represent solely the association and dissociation of the parent radioligand to the receptor. For model overview see Table 1.

(1) $$V_{T}=\;\frac{K_{1}}{k_{2}}\times \left(1+\frac{k_{3}}{k_{4}}\right)$$

(2) $$V_{\mathrm{Tp}}=\frac{K_{1p}}{k_{2p}}$$

(3) $$V_{\mathrm{Tp}}=\frac{K_{1p}}{k_{2p}}\times \left(1+\frac{k_{3}}{k_{4}}\right)$$

**Table 1.** Model overview.

Model	Abbreviation	Fitted parameters
Two tissue compartment model with single input function and fixed *k*_4_	2TSI[*k*_4_]	*V*_B_, *K*_1_, *k*_2_, *k*_3_
One tissue compartment model with dual input function	1TDI	*V*_B_, *K*_1p_, *k*_2p_, *K*_1m_, *k*_2m_
Two tissue compartment model with dual input function	2TDI	*V*_B_, *K*_1p_, *k*_2p_, *k*_3_, *k*_4_, *K*_1m_, *k*_2m_
Two tissue compartment model with dual input function and fixed *k*_4_	2TDI[*k*_4_]	*V*_B_, *K*_1p_, *k*_2p_, *k*_3_, *K*_1m_, *k*_2m_

#### Statistical analysis

The coefficient of variation (COV) was calculated to assess the variability of distribution volumes and obtained by dividing the standard deviation by the mean, of all regions and subjects. The goodness of fit was assessed with the Akaike information criterion (AIC) ^22^ and the reliability of *V*_T_ and *V*_Tp_ by using the percentage standard error (%SE) of the estimates. The fits of dual input models were compared by using *F*-test statistics. The correlations of *V*_Tp_ estimates were assessed by using linear regression analysis and Spearman’s correlation coefficient *r_s_*. A *p*-value below 0.05 was considered significant. Data are presented as mean and standard deviation or median and range, as appropriate.

## Results

### *Ex vivo* radiometabolite analysis

**Figure 1.**
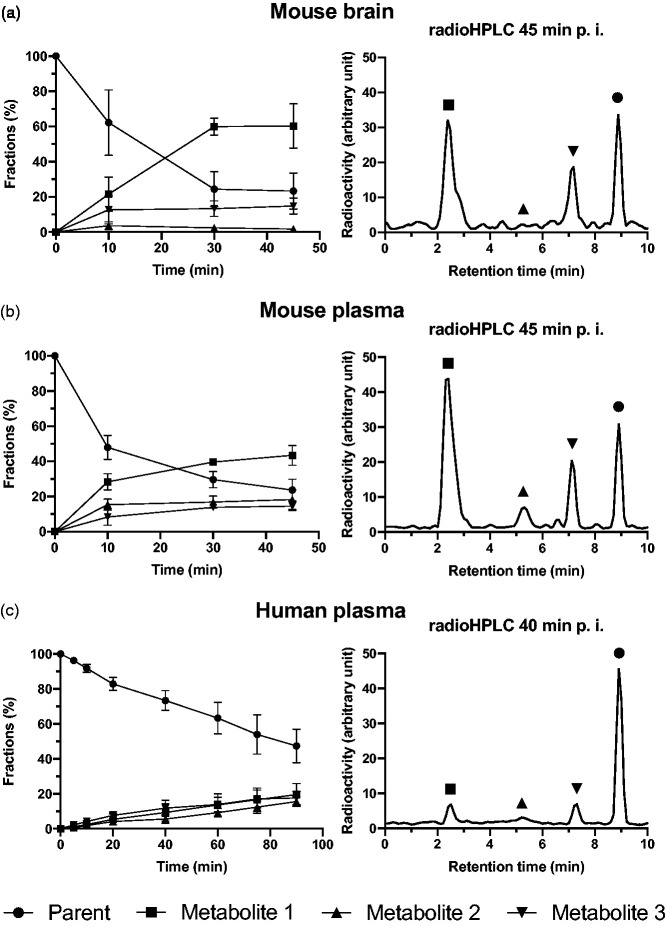
[^11^C]SMW139 radiometabolite analysis. Left panels show mean and standard deviation of parent radioligand and radiometabolite fractions of the whole radioactivity in the sample in mouse brain (a) and mouse plasma (b) (n = 4, 3, and 4, at 10, 30, and 45 min, respectively), and human plasma (c; n = 10). Right panels show exemplary radioHPLC chromatograms from mouse (a, b) and human plasma (c).

There was no exclusion within the small animal study. RadioHPLC showed three major groups of radiometabolite peaks (numbered radiometabolite 1–3 according to the appearance order in radioHPLC chromatogram) with increasing concentrations over time in the plasma of all mice (Figure 1(b)). Only two peaks, the most polar radiometabolite 1 (retention time [Rt] 1.5–3.5 min) and the least polar radiometabolite 3 (Rt 7–8 min), were also clearly present in the mice’s brain homogenates, whereas radiometabolite 2 (Rt 3.5–7 min) was only present in barely quantifiable levels (Figure 1(a)). The mean fraction of unchanged [^11^C]SMW139 at 45 min after injection was similar in plasma and brain: around 23.7 ± 6.2% and 23.3 ± 10.3%, respectively. A high fraction of radioligand metabolites after 10 min (plasma: 52.1 ± 6.8%; brain: 37.8 ± 18.6%) and an apparent transformation into a plateau phase in plasma and brain starting around 30 min p.i. indicates a much faster metabolism of [^11^C]SMW139 in mice compared to humans (Figure 1).

### Human plasma radiometabolites and brain uptake

**Figure 2.**
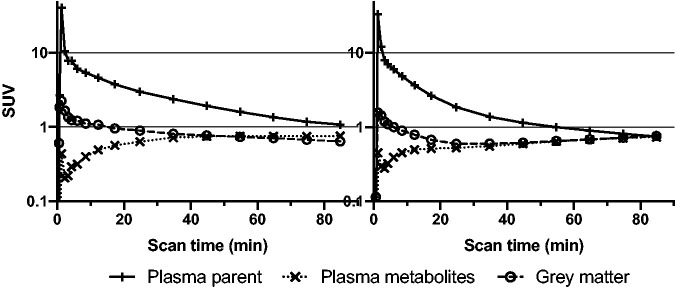
Human time-activity curves. Left panel shows an exemplary finding in one subject with initial peaking followed by washout from the whole-brain grey matter time-activity curves. Right panel shows an exemplary whole-brain grey matter time-activity curve with a slight and steady increase after 20–40 min. Both are depicted with corresponding plasma parent and plasma radiometabolite standardized uptake value (SUV) curves.

Two subjects were excluded due to incomplete blood and plasma data. Plasma radioHPLC measurements showed three groups of radiometabolites peaking with an identical Rt when compared to mice plasma (Figure 1(c)). Human metabolism, however, was slower than that of mice yielding 47.3 ± 9.6% unchanged [^11^C]SMW139 and continuing slightly increasing curves of all three radiometabolites fractions at 90 min p.i. (Figure 1(c)). Visual evaluations of TACs from the whole-brain grey matter showed that five subjects (50%; TU: two subjects, KI: three subjects), after the initial peak during the perfusion and uptake phase, had a clear washout of the tracer until the end of the scan. In contrast, the TACs of the remaining showed a washout up to 20–40 min, followed by a slight and steady increase until the last frame (Figure 2).

### Kinetic modeling

#### Tissue distribution volumes of single and dual input models

**Figure 3.**
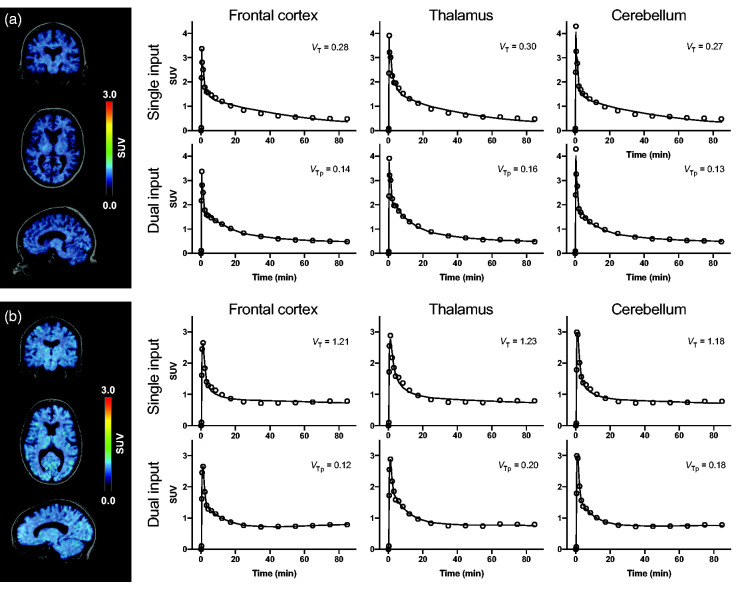
Two tissue compartment model fits with single and dual input function. Summarized standardized uptake value (SUV) images between 40 and 90 min after [^11^C]SMW139 injection and exemplary model fits of two patients with low (a) and high (b) contribution of radiometabolites to brain radioactivity. Abbreviations: *V*_T_, volume of distribution; *V*_Tp_, distribution volume of the parent fraction.

Estimated distribution volumes and %SEs for each evaluated model is presented region-wise in Table 2. 2TDI yielded unstable fits with unreliably high *V*_Tp_ estimates and %SE > 50% in nine (11.3%) out of 80 evaluated regions (eight regions per patient). The observed fitting failures were equally distributed across regions and subjects without a particular VOI that performed poorly. Therefore, these regions were excluded from subsequent comparisons. 2TSI[*k*_4_], 2TDI[*k*_4_], and 1TDI yielded robust fits for all regions with predominantly low %SE < 20% except few outliers (Table 2). None of the regions were excluded. 2TSI[*k*_4_] single input model showed a high COV of *V*_T_ (159.9%), whereas dual input models had a consistently narrow *V*_Tp_ range between 0.04 and 0.24 (COV: 2TDI, 33.3%; 2TDI[*k*_4_], 36.3%; 1TDI, 35.0%; see also Figure 3). *V*_B_ estimates were in close agreement for all evaluated models (*V*_B_ [median, range]: 2TSI[*k*_4_], 0.07, 0.02–0.13; 2TDI, 0.07, 0.02–0.13; 2TDI[*k*_4_], 0.07, 0.01–0.13; 1TDI, 0.07, 0.02–0.13) with a consistently low %SE (median <3.5 for all models).

**Table 2.** Estimated tissue distribution volumes and percentage standard error.

Volume of interest	Two tissue compartment model, single input, *k*_4_ fixed		Two tissue compartment model, dual input^a^		Two tissue compartment model, dual input, *k*_4_ fixed		One tissue compartment model, dual input	
	*V*_T_	%SE *V*_T_	*V*_Tp_	%SE *V*_Tp_	*V*_Tp_	%SE *V*_Tp_	*V*_Tp_	%SE *V*_Tp_
Frontal cortex	0.58 (0.17–10.06)	4.4 (2.8–9.5)	0.14 (0.07–0.21)	8.3 (3.8–13.1)	0.13 (0.07–0.17)	4.9 (2.4–24.0)	0.09 (0.06–0.12)	5.8 (3.3–11.4)
Parietal cortex	0.71 (0.21–10.74)	3.9 (2.2–12.1)	0.15 (0.09–0.20)	13.4 (4.2–32.8)	0.14 (0.07–0.16)	5.5 (2.6–37.7)	0.09 (0.06–0.16)	6.5 (3.7–10.7)
Temporal cortex	0.53 (0.16–8.97)	3.8 (1.7–13.2)	0.13 (0.070.16)	5.9 (2.6–36.3)	0.14 (0.06–0.18)	5.4 (2.3–11.9)	0.10 (0.06–0.16)	4.4 (3.8–12.2)
Caudate	0.43 (0.10–7.24)	5.2 (4.0–7.9)	0.10 (0.04–0.14)	23.0 (6.9–40.4)	0.08 (0.04–0.16)	10.6 (3.5–17.1)	0.07 (0.04–0.12)	7.1 (3.2–20.4)
Putamen	0.61 (0.15–9.20)	6.2 (3.0–15.2)	0.14 (0.07–0.24)	13.6 (4.6–20.1)	0.15 (0.07–0.20)	9.2 (4.4–38.8)	0.10 (0.06–0.14)	6.1 (4.7–13.5)
Thalamus	0.53 (0.13–7.76)	5.5 (3.5–17.4)	0.16 (0.06–0.20)	6.3 (4.7–24.5)	0.14 (0.07–0.24)	6.0 (2.8–21.2)	0.11 (0.07–0.17)	5.4 (2.9–17.6)
Brainstem	0.41 (0.11–6.33)	4.7 (2.8–10.3)	0.14 (0.04–0.20)	9.7 (2.7–32.4)	0.14 (0.05–0.20)	8.3 (3.9–12.6)	0.11 (0.05–0.17)	5.9 (1.9–8.9)
Cerebellar cortex	0.51 (0.15–9.04)	5.0 (2.2–17.4)	0.13 (0.07–0.18)	13.3 (4.2–34.1)	0.11 (0.06–0.18)	5.5 (1.9–23.9)	0.09 (0.05–0.15)	5.0 (3.0–15.5)

Data of healthy volunteers (n = 10) are presented as median and range.

*V*_T_: volume of distribution; *V*_Tp_: distribution volume of unchanged radioligand; %SE: percentage standard error.

^a^Estimates of nine brain regions excluded.

#### Model comparison

Two tissue compartment models showed overall comparable ranges of AIC’s with slight differences between regions. AIC was predominantly smallest for 2TDI[*k*_4_]. Median AIC of 1TDI was tendentially higher than two tissue compartment models (Table 3). Median *F*-value of the comparison between 2TDI and 2TDI[*k*_4_] was below the tabulated value at a *p*-level of 0.05 (*F* critical value: 4.60) for all eight brain regions, suggesting that the simplification of the 2TDI (i.e. using a dual run procedure to fix *k*_4_) can be considered equivalent in terms of the model fit. However, when further simplifying the model to the 1TDI, median *F*-values are predominantly above the critical value (3.74), suggesting that the fit is significantly worsened and thus, this model does not completely describe the pharmacokinetics of [^11^C]SMW139.

**Table 3.** Model fit and model comparison.

Volume of interest	Akaike information criterion				*F*-value		% of regions with better fits than 2TDI^‡^	
	2TSI[*k*_4_]	2TDI	2TDI[*k*_4_]	1TDI	2TDI vs. 2TDI[*k*_4_]*	2TDI vs. 1TDI^†^	2TDI[*k*_4_]	1TDI
Frontal cortex	19.7 (8.5–45.1)	5.0 (–23.1–21.5)	6.0 (–23.7–38.7)	21.4 (–3.9–50.7)	3.0 (–3.1–13.8)	15.3 (–0.4–78.7)	70.0	20.0
Parietal cortex	18.7 (–3.8–58.8)	–0.1 (–33.5–66.9)	14.5 (–9.4–53.4)	27.7 (3.9–63.3)	0.23 (–4.8–138.4)	14.4 (1.9–94.4)	70.0	10.0
Temporal cortex	15.1 (–22.7–53.3)	2.1 (1.8–72.7)	7.9 (–15.7–55.4)	18.1 (–8.2–57.2)	1.3 (–4.6–17.2)	10.8 (–0.8–41.9)	60.0	30.0
Caudate	30.0 (21.4–38.9)	32.9 (13.3–46.7)	32.5 (12.5–59.2)	31.3 (16.5–50.2)	0.12 (–5.0–2.8)	2.8 (–2.2–43.4)	90.0	60.0
Putamen	33.0 (18.9–62.3)	31.0 (0.9–68.6)	32.5 (2.7–51.8)	38.6 (26.4–60.7)	0.0 (–6.2–5.0)	4.6 (0.24–32.7)	90.0	50.0
Thalamus	28.5 (13.5–64.1)	28.0 (1.8–72.7)	18.4 (–1.1–70.5)	32.2 (8.5–67.6)	1.7 (–8.4–6.4)	2.1 (–2.6–46.5)	90.0	60.0
Brainstem	16.0 (–1.2–30.2)	13.4 (–18.0–48.6)	12.8 (–16.8–29.1)	18.4 (–2.5–38.5)	0.11 (–9.5–7.5)	5.6 (–4.5–69.3)	80.0	50.0
Cerebellar cortex	20.7 (–13.1–68.9)	31.9 (–12.1–74.2)	17.8 (–14.9–75.1)	23.2 (–10.2–72.2)	0.31 (–2.4–5.5)	6.5 (1.4–17.4)	90.0	50.0

Data of healthy volunteers (n = 10) are presented as median and range. The tabulated value for *F* statistics at a *p*-level of 0.05 is *4.60 and ^†^3.74, respectively. ^‡^refers to the *F*-test.

2TSI[*k*_4_]: two tissue compartment model with single input function and fixed *k*_4_; 2TDI: two tissue compartment model with dual input function; 2TDI[*k*_4_]: two tissue compartment model with dual input function and fixed *k*_4_; 1TDI: one tissue compartment model with dual input function.

### Correlation analysis

**Figure 4.**
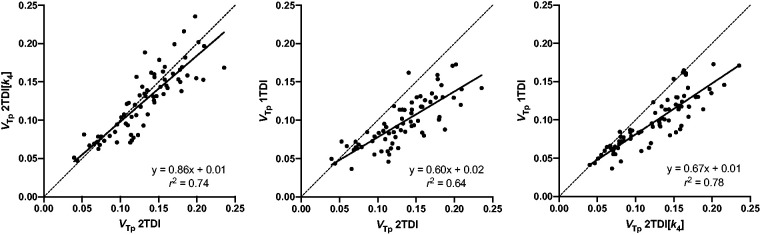
Scatter plots and linear regression analysis. Distribution volume estimates of unchanged [^11^C]SMW139 (*V*_Tp_) within all evaluated brain regions obtained with three models using dual input function: two tissue compartment model (2TDI), two tissue compartment model with a dual run procedure and fixed *k*_4_ (2TDI[*k*_4_]), and one tissue compartment model (1TDI).

Linear regression showed that *V*_Tp_ estimates of all brain regions derived from 2TDI and 2TDI[*k*_4_] (*r*^2^ = 0.74, *p < *0.001) as well as 2TDI[*k*_4_] and 1TDI (*r*^2^ = 0.78, *p < *0.001) strongly correlated with each other, whereas the correlation between 2TDI and 1TDI (*r*^2^ = 0.64, *p < *0.001) was less pronounced (Figure 4). The same applied to each region separately which had high to excellent correlations (2TDI vs. 2TDI[*k*_4_]: *r_s_* = 0.80–1.00, all *p* < 0.01; 2TDI vs. 1TDI: *r_s_* = 0.70–0.88, all *p* < 0.05; 2TDI[*k*_4_] vs. 1TDI: *r_s_* = 0.81–0.94, all *p* < 0.01; Supplemental Table 2).

## Discussion

This study investigating [^11^C]SMW139 metabolism in mice and pharmacokinetics in healthy human participants showed evidence that two [^11^C]SMW139 radiometabolites are present in the brain and must be considered for accurate *in vivo* quantification of [^11^C]SMW139 binding to the human P2X_7_R. Kinetic modeling with plasma parent and radiometabolite input functions allows robust estimation of [^11^C]SMW139 distribution volumes with low variability.

The P2X_7_R is thought to have low expression levels in normal physiology and is upregulated in case of neuronal damage like in neurodegenerative disorders.^6,9^ Since neurodegeneration is a chronic process, neuroinflammatory effects and regulatory mechanisms such as P2X_7_R expression are expected to be subtle.^4,23^ In this context, the correction for possibly confounding, brain-penetrant radiometabolites is of particular importance when evaluating tracers with low *V*_T_ values as it is the case for [^11^C]SMW139.^12,20^

In our cohort of ten healthy volunteers, who were enrolled and examined at two different study sites, kinetic modeling with single input function yielded highly variable *V*_T_ estimates. Furthermore, *V*_T_ estimates in some cases were unexpectedly high and exceeded values that were previously reported with [^11^C]SMW139.^12^ Although the single input model showed robust fitting parameters, the fitted line undershot the late part of the TACs (Figure 3;^12^) suggesting that the assumption of [^11^C]SMW139 radiometabolites not penetrating BBB in humans does not reflect the tracer’s kinetics.

In the presence of three major groups of radiometabolites in the human plasma, however, it is unclear which radiometabolites may enter the brain and hamper the quantification of specific binding of [^11^C]SMW139 to the P2X_7_R. We, therefore, evaluated the [^11^C]SMW139 metabolism in eleven C57BL/6J mice, in order to establish an analogy between humans and mice: considering a faster metabolism in mice as compared to humans,^24,25^ the metabolization of [^11^C]SMW139 is congruent by showing the three [^11^C]SMW139 radiometabolites peaking with the same Rt (Figure 1). The most polar radiometabolite 1 and the least polar radiometabolite 3, were proven to be present in the brain of the mice, whereas radiometabolite 2 was detected only in low amounts in the examined perfused brain homogenates and showed in average around 15% of the fraction levels found in plasma. Notwithstanding the state-of-the-art technique used in perfusion by pumping saline through blood vessels via the mouse’s cardiac chambers after cardiac puncture, the brain may include small residuals of blood and thus, minor fractions of radiometabolite components in the plasma may affect the analysis of brain homogenates. This allows to disregard radiometabolite 2 for kinetic modeling assuming a similarly low ability to penetrate the human BBB.

Consecutively, we adjusted our assumptions for kinetic modeling by accounting for the presence of radiometabolite 1 and 3 within the human brain without specific binding to the P2X_7_R. The variability of *V*_Tp_ estimates was reduced and the 2TDI fits were closer in agreement with the TACs (Figure 3). By reducing the number of fit parameters from seven to six, the precision of *V*_Tp_ values was improved without significantly increasing the residual variation of the fit and by yielding highly correlated *V*_Tp_ estimates. Although a further reduction of the parameters by using the one tissue compartment model resulted in slightly more stable outcome measures, the model fit deteriorated (as indicated by AIC and *F*-test). Furthermore, 1TDI underestimates the radioligand’s *V*_Tp_. The overall small *V*_Tp_ of dual input models seems to be related to a high plasma protein binding of [^11^C]SMW139 (97.2 ± 1.1%), since a low *K*_1p_/*k*_2p_ ratio (Supplemental Table 3) is usually caused by high affinity to plasma proteins. It is also noteworthy that the combination of two metabolites into one input and one tissue metabolite compartment violates the concept of compartment and might impair the model fit to a certain degree. However, modeling with three input functions (by separating *K*_1m_ and *k*_2m_ for both radiometabolites) would – although theoretically more precise – require two additional fitting parameters and therefore be less feasible.

Taken together, the 2TDI[*k*_4_] can be recommended as the preferred model for P2X_7_R quantification with [^11^C]SMW139, though the benefit of adding stability due to fixing *k*_4_ must be confirmed in the respective patient population. The estimation of *k*_4_ is weighted on late time points of the scan and therefore particularly hampered by the observed tracer kinetics with increasing TACs due to brain-penetrant radiometabolites. We initially expected that the consideration of radiometabolites would improve the stability of *k*_4_ such that the added benefit of fixing *k*_4_ is negligible. The combination of low non-displaceable distribution volume and low levels of target expression, however, might not only lead to small *V*_Tp_, but also hamper the stable estimation of “true” *k*_4_. Therefore, adding stability due to reducing the parameters to fit might outweigh variability of *k*_4_ in healthy subjects and patients with subtle neuroinflammatory abnormalities,^4,23^ but a strong inflammatory response accompanied by local heterogeneity of the brain tissue ^26^ due to microglia activation may result in a more pronounced variability of *k*_4_.

Some limitations of this study need to be mentioned. Due to their earlier Rt than the parent tracer in radioHPLC (i.e. greater hydrophilicity), the metabolites are more polar than the parent tracer, and thus, they have a less pronounced ability to cross the BBB. The study does not reveal if radiometabolite 1 crosses the BBB or is metabolized within the brain. To our knowledge, the structure of these radiometabolites is unknown and separate studies are needed to identify the radiometabolites and evaluate the route of metabolism of [^11^C]SMW139.^27^ The radioHPLC analysis of the plasma samples indicates that the metabolism of [^11^C]SMW139 in mice was faster than the metabolism in human subjects. Therefore, it is reasonable to assume that the accumulation rates of radiometabolites 1 and 3 in the human brain are low and might not represent major confounders in this study. The determination of the exact radiometabolite fractions in human subjects was also affected by the low radioactivity concentrations of the samples collected at later time points causing a low signal-to-noise ratio in the radioHPLC analysis. The uncertainty in the estimation of the radiometabolite fractions might have contributed to some variability. However, the kinetic analysis performed using all the plasma radiometabolite fractions (including radiometabolite 2) provided similar results (data not shown), suggesting that the exact determination of the individual fractions did not affect the results to a large extent.

The homogeneous cohort of participants and the combination of data from two study sites with minor methodological differences (see also Supplemental Table 1 and Supplemental Figure 1), make the results fairly generalizable. However, further validation work is required for [^11^C]SMW139 PET. In particular, the model comparison should be validated in patient populations or with an inflammation model in healthy humans or nonhuman primates.^28,29^ In presence of a strong neuroinflammatory response, a simplified pseudo-reference approach for [^11^C]SMW139 is imaginable, assuming no specific binding of the radiometabolites to the P2X_7_R and thus, a similar amount of metabolites in the target and pseudo-reference region. Recent studies with the P2X_7_R radioligand [^11^C]JNJ54173717 did not report an increase of P2X_7_R in patients with Parkinson’s disease or amyotrophic lateral sclerosis.^30,31^ Additional work is ongoing to measure P2X_7_R availability in neurodegenerative disorders using [^11^C]SMW139.

In conclusion, the quantification of the binding of [^11^C]SMW139 to human P2X_7_R is greatly enhanced by using a dual input function model, with the assumption of a similar path of metabolism and BBB penetration ability of radiometabolites in mice and humans. In this cohort of ten healthy volunteers, kinetic modeling with plasma parent and radiometabolite input function allowed robust estimation of [^11^C]SMW139 distribution volumes with low variability.

## Supplemental Material

sj-pdf-1-jcb-10.1177_0271678X221126830 - Supplemental material for Quantification of the purinergic P2X_7_ receptor with [^11^C]SMW139 improves through correction for brain-penetrating radiometabolites Click here for additional data file. Supplemental material, sj-pdf-1-jcb-10.1177_0271678X221126830 for Quantification of the purinergic P2X_7_ receptor with [^11^C]SMW139 improves through correction for brain-penetrating radiometabolites by Joachim Brumberg, Richard Aarnio, Anton Forsberg, Päivi Marjamäki, Vera Kerstens, Mohammad M Moein, Sangram Nag, Saara Wahlroos, Michael Kassiou, Albert D Windhorst, Christer Halldin, Merja Haaparanta-Solin, Patrik Fazio, Vesa Oikonen, Juha O Rinne and Andrea Varrone in Journal of Cerebral Blood Flow & Metabolism
