# Supplementary material for: Quantification of the purinergic P2X7 receptor with [11C]SMW139 improves through correction for brain-penetrating radiometabolites
Source: J Cereb Blood Flow Metab. 2022 Sep 26;43(2):258–68. doi: 10.1177/0271678X221126830 (PMC9903223; doi:10.1177/0271678X221126830)
Supplement: sj-pdf-1-jcb-10.1177_0271678X221126830 - Supplemental material for Quantification of the purinergic P2X7 receptor with [11C]SMW139 improves through correction for brain-penetrating radiometabolites [file sj-pdf-1-jcb-10.1177_0271678X221126830.pdf]

SUPPLEMENTAL MATERIAL

Radiochemistry

Karolinska Institutet. [^{11}C]Methane ([^{11}C]CH_4) was produced in-target via the $^{14}N(p,\alpha)^{11}C$ reaction on nitrogen mixed with 10% of hydrogen, with 16.4 MeV protons using a GEMS PET trace cyclotron (GE, Uppsala, Sweden). Typically, the target gas was irradiated for 15–20 min with a beam current of 35 μA. ^{11}C-Labeled methyl iodide, [^{11}C]CH_3I, was produced following the previously published method [1]. In short, the produced [^{11}C]CH_4 was released from the target and collected in a Porapak Q trap cooled in liquid nitrogen. After collection, the [^{11}C]CH_4 was released from the trap by heating with pressurized air and subsequently [^{11}C]CH_4 was mixed with iodine crystal vapor at 60 °C followed by a radical reaction at 720 °C. The formed [^{11}C]CH_3I was collected in a Porapak Q trap at room temperature and the unreacted [^{11}C]CH_4 was recirculated for 3 min. [^{11}C]CH_3I was released from the Porapak Q trap by heating the trap using a custom-made oven at 180 °C. [^{11}C]CH_3OTff was produced by online transfer of [^{11}C]CH_3I through a glass column packed with silver triflate at 165 °C. Carbon-11 labelled [^{11}C]SMW139 was obtained by trapping [^{11}C]CH_3OTff at room temperature in a reaction vessel containing the desmethyl precursor SMW167 (2-chloro-5-hydroxy-N-(((3s,5s,7s)-3,5,7-trifluoroadamantan-1-yl)methyl) benzamide), (0.5 – 1 mg, 1.3 μmol-2.6 μmol) and NaOH (0.5M, 3 μL) in acetone (400 μL). The reaction mixture was diluted with sterile water (500 μL) before injecting to the built-in high-performance liquid chromatography (HPLC) system for the purification of the desired radiolabelled product. The HPLC system consisted of a semi-preparative reverse phase XBridge column (C18, 10 × 250 mm, 5 μm particle size) and a Merck Hitachi UV detector (λ = 254 nm) (VWR, International, Stockholm, Sweden) in series with a GM-tube (Carroll-Ramsey, Berkley, CA, USA) used for radioactivity detection. Acetonitrile / 0.1% Trifluoroacetic acid (TFA), 50:50 (v/v) was used as HPLC mobile phase with a flow rate of 6 mL/min. The radioactive fraction corresponding to pure [^{11}C]SMW139 was collected from HPLC and evaporated to dryness. The final purified [^{11}C]SMW139 was formulated in 6–mL phosphate buffered saline (pH7.4) and the formulated product was then sterile filtered through a Millipore Millex® GV filter unit (0.22 μm) for further use in vivo.

Turku PET Centre. Radiochemistry was performed as previously described [2].

Quality control and molar activity (MA) determination.

Karolinska Institutet. The radiochemical purity, identity, and stability of [^{11}C]SMW139 was determined by an analytical HPLC system which included an XBridge RP column (C18, 4.6µm × 150 mm,), Merck Hitachi L-7100 Pump, L-7400 UV detector and GM-tube for radioactivity detection (VWR International). The mobile phase CH_3CN/H_3PO_4 (0.01M aq. solution) with an isocratic HPLC method (43:57) and flow rate of 2 mL/min was used to elute the product. The effluent was monitored with a UV absorbance detector (ƛ = 230 nm) coupled to a radioactive detector (b-flow, Beckman, Fullerton, CA). The retention time (Rt) of [^{11}C]SMW139 was 4–5 min. The identity of [^{11}C]SMW139 was confirmed by using HPLC with the co-injection of the authentic non-radioactive SMW139 (2-chloro-5-methoxy-N-(((3s,5s,7s)-3,5,7-trifluoroadamantan-1-yl)methyl) benzamide) standard. The MA of the final product was measured by analytical HPLC which included XBridge RP column (C18, 4.6µm × 150 mm,), Merck-Hitachi L-7100 Pump, L-7400 UV detector. The mobile phase CH_3CN/H_3PO_4 (0.01M aq. solution) with an isocratic HPLC method (46:54) and flow rate of 2 mL/min. MA was calibrated for UV absorbance (ƛ = 230 nm) response per mass of ligand and calculated as the radioactivity of the radioligand (GBq) divided by the amount of the associated carrier substance (µmol). Each sample was analysed three times and compared to a reference standard also analysed three times.

Turku PET Centre. The radiochemical purity, identity, stability and the MA of [^{11}C]SMW139 was determined as previously described[2].

Small animal study

Radio detector high-performance liquid chromatography analysis. The supernatant was injected into the radioHPLC (Merck Hitachi L-7100 gradient pump system coupled with a Radiomatic 150 TR, Packard, USA) using a 1 mL loop which was filled with distilled water to minimize the acetonitrile composition prior to the injection. The loop and Hamilton injection syringe were rinsed three times with 1.0 mL 1:1 (v/v) acetonitrile:water solution between each injection to remove any residuals from previous samples. All samples were analysed using a

5.0 mL/min eluent flow, Phenomenex Luna 5µm C18(2) 100 Å 250x10 mm column and with the following method: a gradient of acetonitrile (A), 0.1 % Trifluoroacetic acid in water (B) was used: 0–8.0 min (A/B) 30:70 → 90:10, 8.0–9.0 min (A/B) 90:10, 9.0–10.0 min (A/B) 90:10 → 30:70, 10.0–12.0 min (A/B) 30:70. A radioactive standard was prepared by spiking 65:35 (v/v) acetonitrile:water with [^{11}C]SMW139 (~3 kBq/mL) and injected into the same system to confirm the Rt of the unmetabolized [^{11}C]SMW139. RadioHPLC data were analysed using Merck Hitachi D-7000 HSM software (Merck, Darmstadt, Germany). Radio-chromatograms were integrated, decay corrected with the Rt of the peak, and the amount of parent compound and radioactive metabolites were calculated as a percentage of the total of the areas of all detected radioactive fractions.

Human study

Arterial plasma input for [^{11}C]SMW139 and radioactive [^{11}C]SMW139 metabolites. At Karolinska Institutet, the individual blood curve over the first 5 min from the ABSS was merged with the curve from manual blood samples. Radioactivity concentrations in plasma and blood were divided yielding a plasma/blood ratio curve. Using linear interpolation, the plasma/blood ratio curve was extrapolated from 0 s to the end of sampled ABSS data. Plasma time-activity curves covering the whole scan were generated by multiplying the extrapolated plasma/blood ratio curve with the ABSS blood curve and by fusing the result with the plasma curve from manual samples. At Turku PET Centre, input processing assumed that parent radioligand concentration in blood cells is zero during the time of ABSS data collection. Plasma time-activity curves covering the whole scan were generated by dividing the ABSS blood curve by (1 - hematocrit) and by merging the result with the plasma curve from manual samples. Subsequent processing was performed concordantly for both study sites with PMOD (version 3.7, PMOD Technologies LLC). Data for parent fraction of [^{11}C]SMW139 were fitted using a mono-exponential fit and were multiplied with the uncorrected plasma time-activity-curve to obtain the parent input curve (i.e. the plasma radioactivity of unchanged [^{11}C]SMW139). Furthermore, we estimated an input curve representing the activity of presumably brain-penetrant [^{11}C]SMW139 radiometabolites in the plasma. Therefore, the parent fraction and the

fraction of the radiometabolite, which was assumed not to be present within the brain, were combined, fitted with a mono-exponential function and multiplied with the plasma time-activity-curve. The obtained curve was subsequently subtracted from the plasma input curve, which resulted in the radiometabolite input curve. Finally, blood and plasma input curves were delay corrected.

References

1. Andersson J, Truong P, Halldin C. In-target produced [11C]methane: Increased specific radioactivity. *Appl Radiat Isot.* 2009;67:106-110.

2. Aarnio R, Alzghool OM, Wahlroos S, et al. Novel plasma protein binding analysis method for a PET tracer and its radiometabolites: A case study with [(11)C]SMW139 to explain the high uptake of radiometabolites in mouse brain. *J Pharm Biomed Anal* 2022; 219: 114860.

SUPPLEMENTAL TABLE 1. Differences of data acquisition and processing methods

	Imaging procedures	
	Karolinska Institutet	Turku PET Centre
MR system	GE Healthcare Discovery MR750	Philips Ingenuity TF PET/MR
PET reconstruction, matrix size	256 x 256 x 207	200 x 200 x 150
	Arterial blood sampling and input processing	
	Karolinska Institutet	Turku PET Centre
ABSS duration	10 min	5 min
Manual blood samples	2 mL at ~ 2, 5, 10, 20 min, and 4 mL at ~ 40, 60, 75, and 90 min p.i.	2 mL at ~ 5, 10, 20, 40, 60, 75, and 90 min p.i.

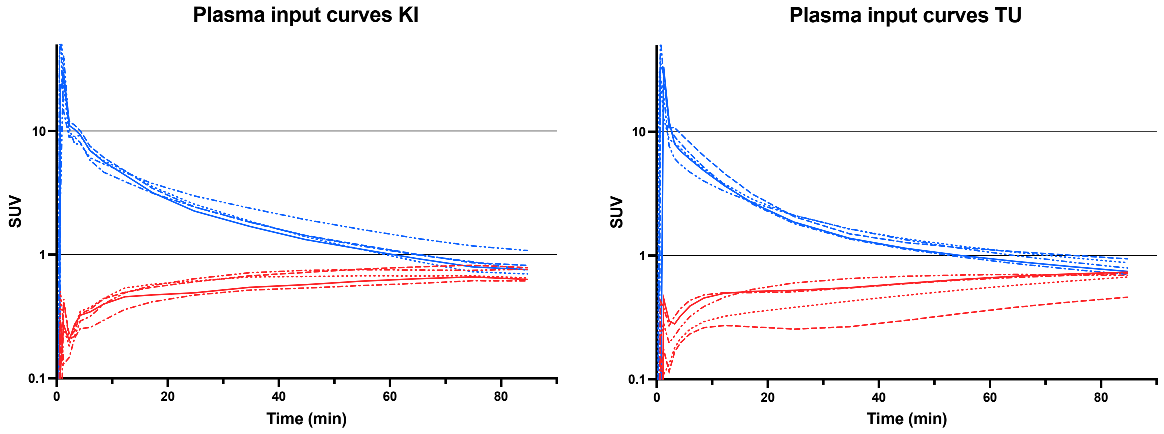

SUPPLEMENTAL FIGURE 1 Plasma parent and metabolites input curves. Plasma parent (blue) and metabolites (red) input curves of each participant enrolled at the Karolinska Institutet (KI; left panel) and at Turku PET Centre (TU; right panel). Line patterns indicate the corresponding plasma and metabolites input curves for each participant.

SUPPLEMENTAL TABLE 2. Correlation analysis

Volume of interest	2TDI vs. 2TDI[k_4]		2TDI vs. 1TDI		2TDI[k_4] vs. 1TDI	
	r_s	*p*-value	r_s	*p*-value	r_s	*p*-value
Frontal cortex	0.95	<0.001	0.70	0.036	0.81	0.005
Parietal cortex	0.82	0.007	0.88	0.002	0.89	<0.001
Temporal cortex	0.92	<0.001	0.82	0.007	0.92	<0.001
Caudate	0.98	<0.001	0.71	0.047	0.87	0.001
Putamen	0.90	<0.001	0.78	0.013	0.82	0.004
Thalamus	1.00	<0.001	0.87	0.003	0.89	<0.001
Brainstem	0.80	0.010	0.85	0.004	0.94	<0.001
Cerebellar cortex	0.85	0.004	0.83	0.005	0.84	0.002

n = 10 healthy volunteers. Abbreviations: 2TDI, two tissue compartment model with dual input function; 2TDI[k_4], two tissue compartment model with dual input function and fixed k_4; 1TDI, one tissue compartment model with dual input function.

SUPPLEMENTAL TABLE 3. Estimates of blood volume and *k*-ratios for two tissue compartment model, dual input, k_4 fixed

Volume of interest	V_B	%SE V_B	K_{1p}/k_{2p}	%SE K_{1p}/k_{2p}	k_3/k_4	%SE k_3/k_4	K_{1m}/k_{2m}	%SE K_{1m}/k_{2m}
Frontal cortex	0.06<br>(0.03-0.11)	2.0<br>(1.0-16.2)	0.06<br>(0.04-0.10)	6.5<br>(2.8 -11.4)	0.70<br>(0.28-2.65)	13.9<br>(4.2-45.6)	0.92<br>(0.57-1.35)	5.7<br>(2.0-23.4)
Parietal cortex	0.07<br>(0.03-0.13)	2.5<br>(1.3-8.5)	0.06<br>(0.02-0.12)	6.6<br>(3.7-18.6)	1.17<br>(0.01-3.43)	15.8<br>(10.1-295.3)	1.04<br>(0.68-1.55)	5.8<br>(3.4-14.6)
Temporal cortex	0.06<br>(0.04-0.12)	1.9<br>(0.8-11.3)	0.06<br>(0.03-0.14)	8.8<br>(2.9-25.2)	0.97<br>(0.19-1.70)	17.8<br>(8.2-104.4)	0.84<br>(0.60-1.33)	6.0<br>(4.1-20.8)
Caudate	0.05<br>(0.04-0.08)	3.8<br>(2.0-14.3)	0.05<br>(0.01-0.11)	10.3<br>(5.8-100.4)	0.45<br>(0.10-3.37)	47.6<br>(22.7-110.7)	0.79<br>(0.38-1.20)	8.9<br>(4.8-25.4)
Putamen	0.07<br>(0.01-0.11)	3.7<br>(1.7-94.8)	0.07<br>(0.06-0.11)	8.2<br>(4.2-13.5)	0.82<br>(0.23-1.80)	20.8<br>(13.2-88.1)	0.92<br>(0.52-1.67)	13.5<br>(5.4-44.9)
Thalamus	0.07<br>(0.01-0.13)	3.4<br>(1.3-50.9)	0.07<br>(0.05-0.13)	8.6<br>(4.0-19.3)	0.43<br>(0.18-1.53)	25.8<br>(7.5-221.2)	0.78<br>(0.52-1.45)	12.2<br>(4.0-25.8)
Brainstem	0.06<br>(0.03-0.11)	1.9<br>(1.2-8.6)	0.07<br>(0.03-0.16)	11.9<br>(5.7-28.4)	0.99<br>(0.01-3.32)	34.9<br>(5.8-195.8)	0.74<br>(0.32-0.96)	8.4<br>(2.8-22.4)
Cerebellar cortex	0.08<br>(0.04-0.13)	2.8<br>(1.0-6.3)	0.06<br>(0.04-0.10)	9.5<br>(3.9-36.2)	0.63<br>(0.12-2.85)	35.2<br>(4.9-163.4)	0.96<br>(0.55-1.33)	8.5<br>(3.0-30.8)

Data of healthy volunteers (n = 10) are presented as median and range. Abbreviations: %SE, percentage standard error.
